# Characterization of lightning-induced overvoltages in wind farms

**DOI:** 10.1371/journal.pone.0325514

**Published:** 2025-06-10

**Authors:** Tamer Eliyan, Saad F. Al-Gahtani, Z.M.S. Elbarbary, Fady Wadie

**Affiliations:** 1 Department of Electrical Engineering, Faculty of Engineering at Shoubra, Benha University, Cairo, Egypt; 2 Department of Electrical Power and Machines Engineering, The Higher Institute of Engineering at El-Shorouk City, Alshorouk Academy, Cairo, Egypt; 3 Department of Electrical Engineering, College of Engineering, King Khalid University, Abha, Saudi Arabia; 4 Center for Engineering and Technology Innovations, King Khalid University, Abha, Saudi Arabia; 5 Mechatronics and Robotics Engineering Department, Faculty of Engineering, Egyptian Russian University, Badr City, Egypt; King Fahd University of Petroleum & Minerals, SAUDI ARABIA

## Abstract

Wind farms are exposed to various weather hazards, including lightning strikes, which can pose significant risks. However, the impact of different wind farm topologies on the magnitude of lightning-induced overvoltages has not been extensively studied, creating a gap in existing literature. This paper addresses this gap by analyzing the characteristics of lightning-induced overvoltages injected into the grid for various wind farm topologies. The scientific scope of this study is to evaluate the influence of wind farm topology on the severity of different types of lightning-induced overvoltages including positive, negative, and double-peaked lightning strikes, using simulation-based analysis. The topologies tested include radial, single-sided ring (SSR), double-sided ring (DSR), and star topologies. The results demonstrate that radial topology leads to the highest overvoltage injection, while switching to SSR, DSR, or star topologies results in reductions of overvoltage by 11.5% to 51.0%, 39.5% to 66.0%, and 62.3% to 89.0%, respectively. These results support a topology-based risk assessment approach, offering clear guidance for selecting configurations that improve lightning resilience.

## Introduction

The transition from fossil-fuel based energy generation to green energy based resources has been the main occupying challenge facing researchers. The challenge is inherited due to non-uniform nature of the generated power by renewable energy sources in addition to their limitation to certain geographical locations [1–4]. Such a tie to specific locations was the reason for renewable energy resources such as wind farm to be scattered over large terrain exposed to hazards of random weather conditions. This hazardous situation for wind farms mandated protective measures to be taken for the protection of wind farms against weather conditions [5–10].

To review the protection of wind farms against lightning strikes, this paper begins by exploring the vulnerability of key components, starting with the tower structure, and then examines how lightning-induced overvoltages propagate through different wind farm topologies. The towers of wind farms are particularly vulnerable to lightning strikes due to their height and exposed nature, with the blade being the most likely part to be struck, given its elevated position atop the tower [11–15]. A lightning strike on the blade can cause significant electrical disturbances, leading to a rise in potential across various tower elements [13–16]. Previous studies have focused on the impact of lightning strikes on the tower structure [17–20] and the grounding system [12,21,22].

Beyond direct strikes, lightning-induced overvoltages can propagate through the wind farm, potentially affecting other network components if the overvoltage exceeds the protection level provided by the lightning protection system [23–27]. While prior research has primarily focused on surge propagation in radial wind farms [23–27], it has largely overlooked how these overvoltages behave in other topologies.

This paper addresses this gap by investigating lightning-induced overvoltage propagation across different wind farm topologies. Understanding how these overvoltages spread is critical for assessing the vulnerability of each configuration. Four widely accepted topologies—radial, single-sided ring (SSR), double-sided ring (DSR), and star—are examined [28–29]. The study evaluates how overvoltage propagation varies in each topology and the effectiveness of their respective lightning protection strategies, ultimately identifying the most reliable configuration for safeguarding wind farms from lightning strikes.

To ensure the comprehensibility of the paper, various types of lightning strikes were included in the study. Commonly employed lightning types include positive, negative and double peaked lightning strikes [30–33]. The study was performed upon ATP/EMTP simulation platform. The novel contributions of this paper are as follows:

1. The paper investigates the rarely studied behavior of lightning-induced overvoltages across various wind farm topologies, filling a gap in the existing literature.
2. It extends the research by considering multiple types of lightning strikes, offering a more detailed and holistic understanding of their effects.
3. The paper extracts and highlights the distinctive features of lightning-induced overvoltages injected into the grid, taking into account different wind farm configurations and lightning types.
4. It evaluates the influence of wind farm topology on the magnitude of overvoltages injected into the grid, identifying the most reliable topologies in terms of performance during lightning strikes. This evaluation aids design engineers in making more informed decisions when selecting the optimal wind farm topology during the design phase.

The rest of this paper is arranged in the following order. In “System under study”, the data of the modeled system is presented. The modeling process of the system is presented in “Modeling of the system”. The results of simulation process and the observed characteristic of the lightning induced overvoltage in each wind farm topology are provided in “Simulation results”. The results are analyzed and discussed in “Analysis and discussion of simulation results”. Finally, conclusions are drawn in “Conclusion”.

## System under study

**Fig 1:**
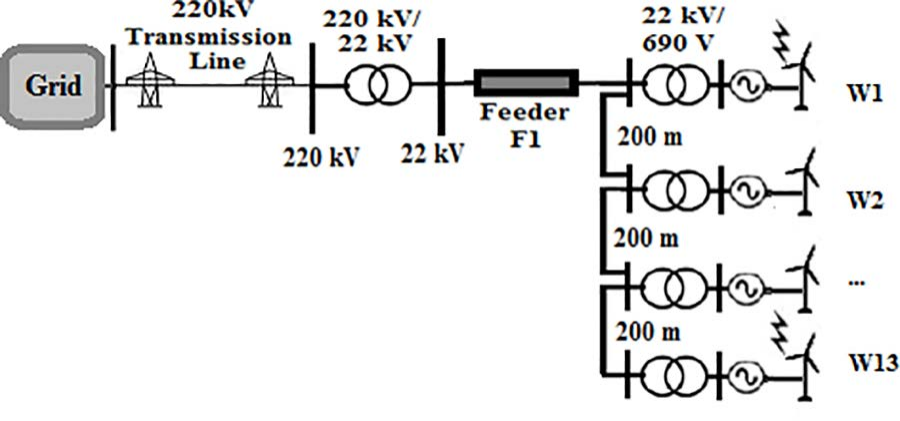
Topology of radial wind farm.

**Fig 2:**
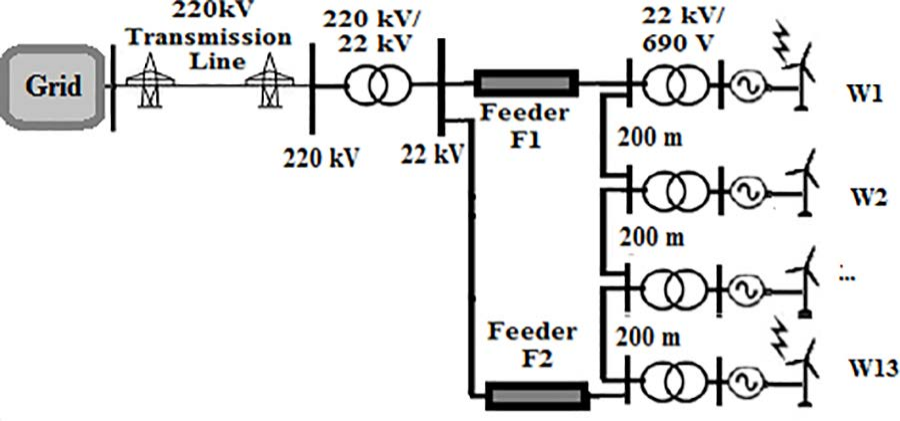
Topology of single sided ring wind farm.

**Fig 3:**
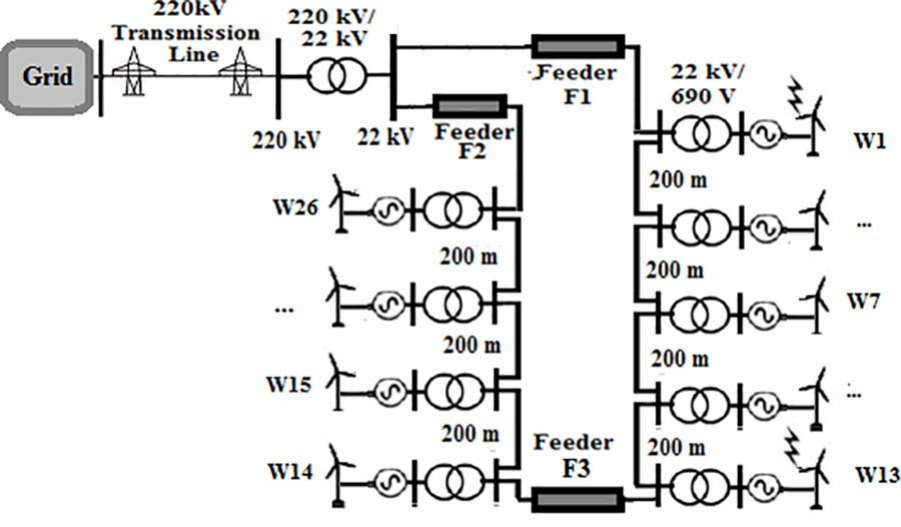
Topology of double sided ring wind farm.

**Fig 4:**
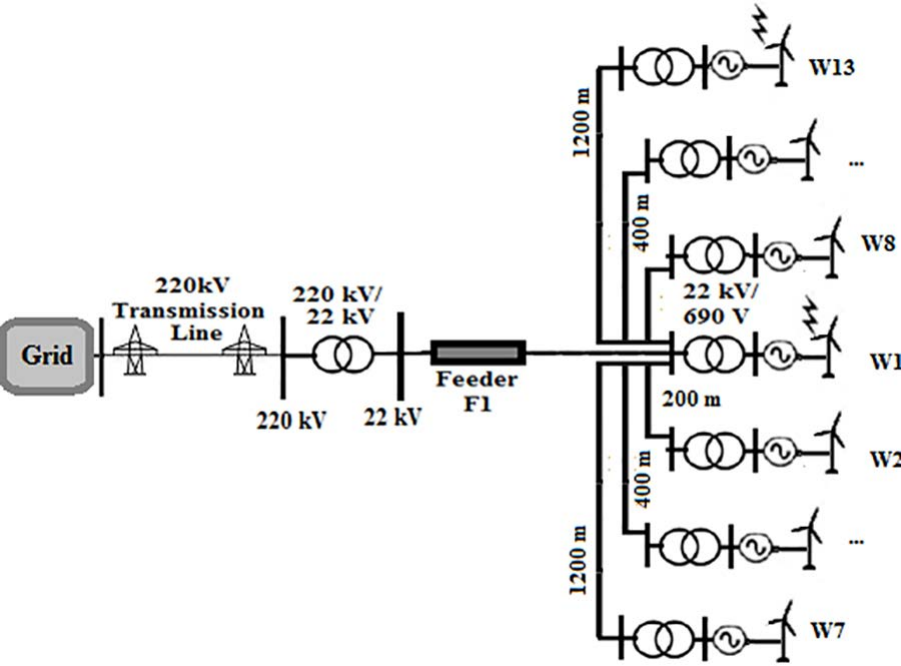
Topology of star wind farm.

A 550 MW real wind farm system located in Zaafrana, Egypt was selected to be under study. The consists of 700 identical wind turbines with a 1 MVA 690 V/22 kV-transformers connected to each of them. Each two turbines in sequential order are connected by Cables of 200-m length. The farm supplies power to the gird through a 220/22 kV substation. Since this study aims to examine the impact of change the topology of the farm, the connection of the turbines will be changed to type of the topology under study while keeping the characteristic features of the elements of the farm unchanged. The four topologies that will be tested radial, SSR, DSR and star topologies are shown in Figs 1–4. Feeders F1 has a length of 8 km for all topologies. Feeder F2 is set to 10.4 km in SSR topology and 6.5 km in DSR topology. Feeder F3 has a length of 1 km. Even though that it is mentioned earlier that the length of the cables in between two series turbines is 200 m, that rule would not be applicable to star topology for its inherited nature that all turbines are connected to a certain node. The connection is simplified as W1 is at the center of the topology, with W2 to W7 arranged on one side and W8 to W13 on the other side. For such reason, the length of the cables in star connection will be as follows; 200 m for W2, 400 m for W3, 600 m W4 and so on till W7 and the same sequence is used for W8 to W13. The modifications done is based upon those presented in [34]. The modeling of the elements of the system is discussed in the next section.

## Modeling of the system

### Modeling of the wind turbine

#### Tower and blades of the wind turbine.

The blades of the wind turbine and tower body are modeled based on their physical characteristics. The surge impedance of a blade was approximated from conical equations based upon electromagnetic ﬁeld theory as shown in (1). While the surge impedance of the tower was approximated from cylindrical equation as given in (2) [35,36].


$$Z_{t}=60\;ln(\frac{\sqrt{2}\;H}{r})$$ (1)

$$Z_{B}=60\;ln(\frac{2\;L_{b}}{r_{b}})$$ (2)

Where (H) and (r) are height and radius of the base of the tower of the wind turbine respectively. $L_{b}$ and $r_{b}$are the length of the blade and the radius of down conductor within the blade of the turbine. It should be mentioned that more detailed modeling methods are available in literature [1–8] but these publications were focused on the impact of lightning on the tower and grounding systems. However, the main focus of this study is the impact of the overvoltage induced by into the wind turbine electrical power system and hence to the topology of the wind farm that is intended to be studied in this paper. For this reason, the usage of simplified but still reasonable sound modeling used is acceptable as in [35,36].

#### Grounding resistance of the tower.

The challenge in modeling the grounding resistance of the tower is the variation of its value during transient response. This change in its value during lightning strikes is due to soil ionization caused by excessive energy associated with the lightning current. The modeling of the grounding resistance could be represented as a non-linear resistance (Rg) with value as given in (3) [35,36].


$$\begin{array}{c} R_{g}=\left\{ \;\;\;\;\begin{array}{c} {\;\text{R}}_{\text{go}}\;\;\;\;\;\;\;\;\;\;(I<{\text{I}}_{\text{o}})\;\;\;\;\;\\ \;\;\;\;\frac{{\text{R}}_{\text{go}}}{\sqrt{1+\;\frac{\text{I}}{{\text{I}}_{\text{o}}}}}\;\;\;\;\;\;\;\;\;\;\;\;\;\;(I>{\text{I}}_{\text{o}}) \end{array}\right.\;\; \end{array}$$ (3)

$$I_{0}=\;\frac{\rho_{s}\;E_{o}\;}{2\pi \;R_{go}^{2}}$$ (4)

where $\begin{array}{c} R_{g},\;R_{go} \end{array}$ are the calculated value of the grounding resistances of the tower and grounding resistance of the tower during normal operating conditions, I represents the current flowing through the grounding resistance and $I_{o}\;$ is the minimum value current required for soil ionization that could be calculated from (4). $E_{o}$ and $\rho_{s}$ are the gradient of soil ionization and frequency dependent soil resistivity respectively. The values of $\begin{array}{c} E_{o},\;\rho_{s} \end{array}$ and $R_{go}$ were defined according to [37] to be 400 kV/m, 1000 Ω.m and 10 Ω respectively. In ATP, a non-linear resistance model that could be controlled through mathematical formulations is available via TACSRES component.

#### Generator of the wind turbine.

The generator within the wind turbine was modeled using 690 V synchronous generator model. The leakage reactance of the generator is 0.1 H [16]. A stray capacitance is formed between the tower and generator in terms of few nano-farads which was set to 10 nF in this study [35,36]. Such capacitance is highly important to be modeled since it allows simulate the induction of the overvoltage caused by lightning into the electrical system of the wind farm.

#### Transmission lines, feeders and cables.

Frequency-dependent model was used in modeling transmission lines with their parameters given in Table 1 as articulated in [34]. Similarly, frequency-dependent cable model was used for modeling of cables with their lengths as given in section 2.

**Table 1:** Transmission line parameters.

	Positive and negative sequence parameters	Zero sequence parameters
Resistance (Ω/km)	0.03	0.13
Reactance (Ω/km)	0.306	0.83
Susceptance (mS/km)	3.25	2.3

#### Power transformers.

To accurately model the transformer during transient analysis, a frequency-dependent transformer model is used and the effect of the stray capacitance is considered between each winding and the ground and the capacitance between the windings with each other. These capacitances were modeled across the main transformer model using capacitive elements connected across transformer component upon ATP [16,34].

#### Lightning strike.

The impulsive nature of the lightning strike has generally been modeled as an impulse current surge that is injected to the strike location through a current source. The characteristic of lightning surge is formulated according to Heidler function as given in (5) and (6) [23,30,38].


$$i(t)=\;\frac{I_{o}}{\eta }\;.\;\frac{(\frac{t}{\tau_{1}}{)}^{n}}{1+\;(\frac{t}{\tau_{1}}{)}^{n}}\exp\left(-\;\frac{t}{\tau_{2}}\right)$$ (5)

$$\eta =\exp(-\left(\frac{\;\tau_{1}}{\tau_{2}}\right)(n\;\frac{\;\tau_{2}}{\tau_{1}}{)}^{\frac{1}{n}}\;)$$ (6)

where $I_{o}$ is the peak value of lightning impulse; $\tau_{1}$ and $\tau_{2}\;$are the rise tail times of the impulse respectively, n is the exponent factor and η is the peak value correction factor. The values of the parameters of the lightning impulse depend on its type. Three types are studied in this work; positive, negative and real-life recorded double peaked lightning strikes at Mount San Salvatore (MSS) and Morro do Cachimbo (MCS) stations. The front and tail times for these types are set as follows:

1. 10/350 µs for positive lightning strikes that is based upon the IEC 61400−24 and IEC 61643−11 standards [39,40].
2. 1/200 µs for negative lightning strikes that was based upon the IEC 61400−24 standard [39].
3. A superposition of seven Heidler functions were used to recreate the double peaked lightning strikes recorded at MSS and MCS substations [41]. The parameters of these seven impulses for MCS and MSS are presented in Table 2.

**Table 2:** Parameters of double peak lightning strikes.

Curve #	Recorded at MSS				Recorded at MCS			
	$I_{P}$	n	$\tau_{1}$	$\tau_{2}$	$I_{P}$	n	$\tau_{1}$	$\tau_{2}$
1	3	2	3	76	6	2	3	76
2	4.5	3	3.5	25	5	3	3.5	10
3	3	5	5.2	20	5	5	4.8	30
4	3.8	7	6	60	8	9	6	26
5	13.6	44	7.6	60	16.5	30	7	23.2
6	11	2	100	600	17	2	70	200
7	5.7	15	11.7	48.5	12	14	12	26

## Simulation results

The modified windfarm topologies of Zaafrana system were modeled as described in section 3 and lightning was created to hit the blades of the wind turbine. As the aim of this research work is to evaluated the impact of changing the wind farm topology on the induced lightning overvoltage that would reach the grid, two scenarios were assumed for the lightning strike events. The first scenario assumes the lightning strikes the blade of the tower closest to the grid while the second scenario assumes that lightning strikes the two farthest to the grid. These two scenarios were selected as they represent the extreme possible cases of lightning strikes from the closest to the farthest tower. For each scenario, the overvoltages propagating from blade to tower and then to the 220 kV/22 V transformer connected to the grid will be examined. These scenarios will be applied to each wind farm topology as follows.

### Radial topology

**Fig 5:**
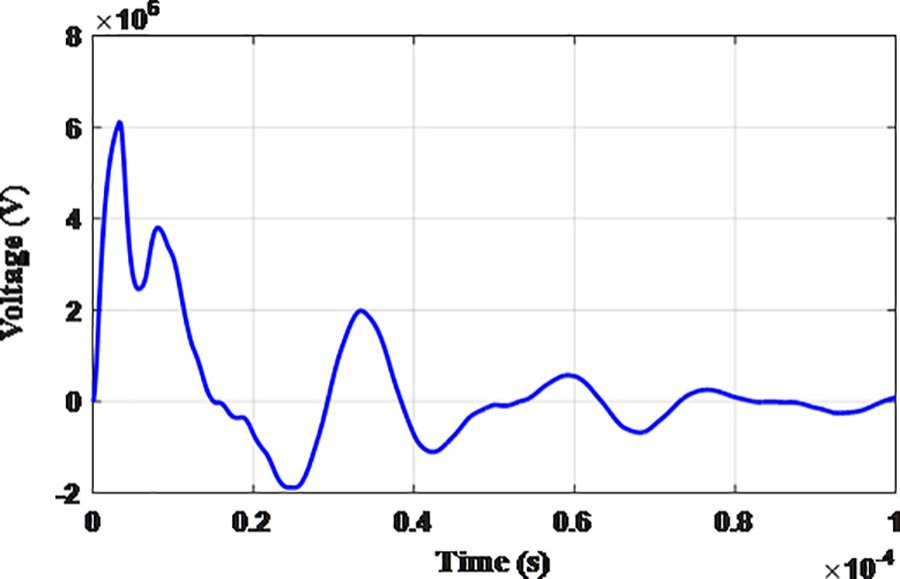
Overvoltages for case R-1 at tip of the blade of the turbine.

**Fig 6:**
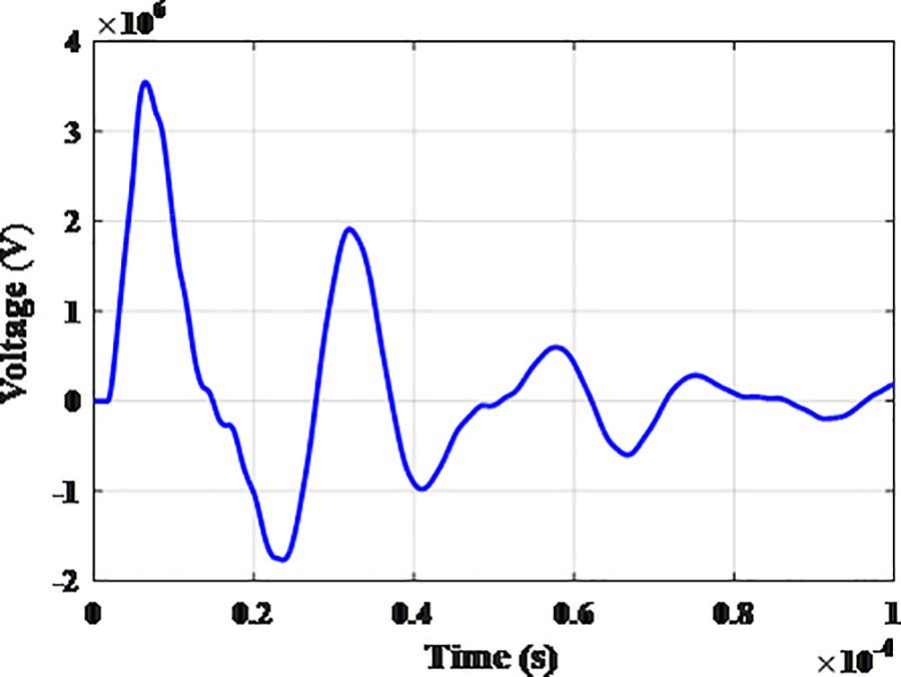
Overvoltages for case R-1 at the top of tower.

**Fig 7:**
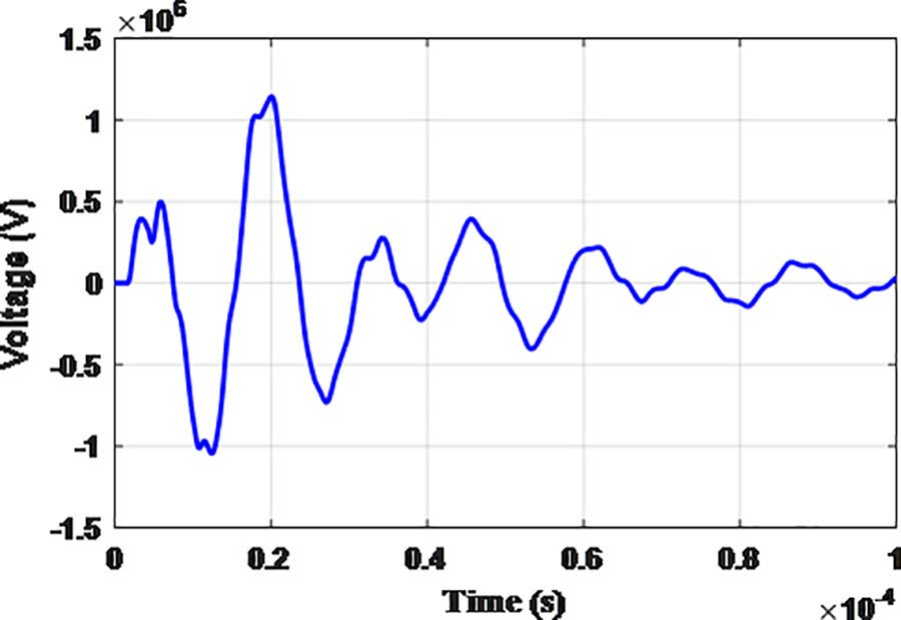
Overvoltages for case R-1 at the generator inside the turbine.

**Fig 8:**
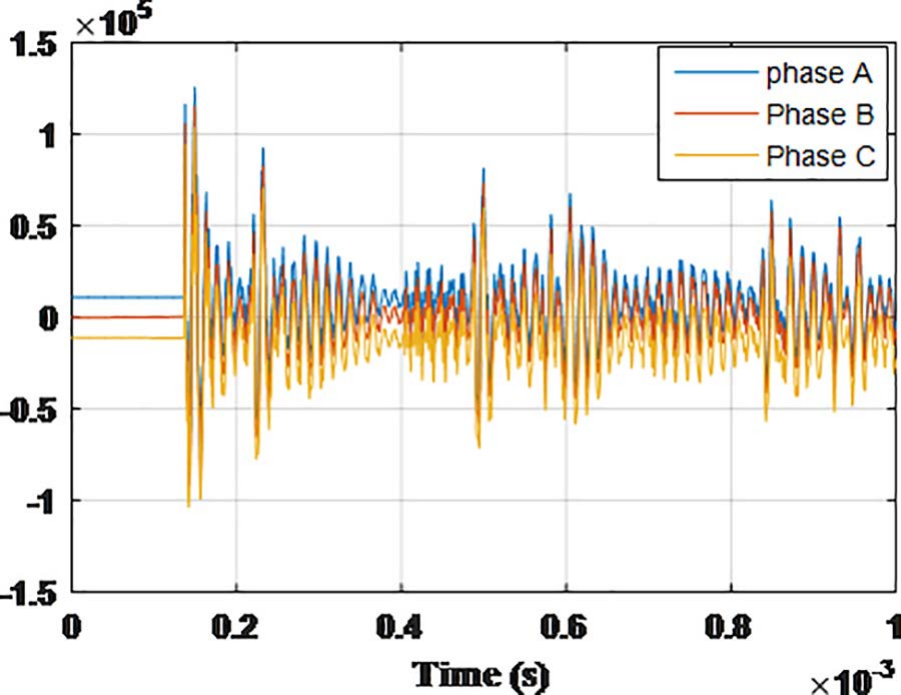
Overvoltages for case R-1 at the Low-voltage bus of the grid connected transformer.

In this topology, W1 is the turbine closest to the grid while W13 is the farthest one. The two scenarios will be considered that lightning once strikes W1 and in the second scenario lightning strikes W13. Firstly, a 50 kA, 10/350 µs positive lightning strikes are considered in both scenarios. 50 kA Negative strikes in addition to MSS and MCS with current peaks from Table 2 were also considered to ensure maximum coverage for the expected overvoltages. Therefore, a total of 8 cases are studied in radial topology. The first case for positive strikes hitting W1 will be named R-1 where stands for radial and 1 represents number of the first case in which positive strike hits W1. Second case will be named R-2 where positive strikes hits W13. The remaining cases are named from R-3 to R-8 as described in Table 3. The overvoltage induced at each cases upon the blade tip, the top of the tower, the generator within the turbine and the 22 kV bus of the 220/22 kV transformer are recorded in Table 3. The overvoltage at the blade tip, top of the tower, generator and 22 kV bus of the Grid connected transformer are shown in Figs 5–8.

**Table 3:** Maximum overvoltages recorded for radial topology.

Case name	Turbine hit	Type and parameters of lightning	Maximum Voltage (MV)			Maximum Voltage at low voltage bus of grid-connected transformer (kV)		
			Blade tip	Top of tower	Generator	Phase A	Phase B	Phase C
R- 1	W1	Positive 50 kA, 10/350 µs	6.11	3.54	1.12	125.02	115.39	103.80
R- 2	W13	Positive 50 kA, 10/350 µs	6.11	3.53	1.11	177.55	167.43	155.16
R- 3	W1	Negative 50 kA, 1/200 µs	6.69	4.40	1.39	470.41	459.98	448.46
R- 4	W13	Negative 50 kA, 1/200 µs	6.69	4.35	1.33	273.42	263.30	251.48
R- 5	W1	MCS, parameters from table 1	3.86	2.39	0.71	71.35	61.49	73.06
R- 6	W13	MCS, parameters from table 1	3.86	2.39	0.69	108.66	98.55	86.70
R- 7	W1	MSS, parameters from table 1	3.23	1.72	0.49	63.31	52.90	52.14
R- 8	W13	MSS, parameters from table 1	3.23	1.71	0.47	86.17	76.07	64.22

From the results shown in Table 3, several key features could be extracted for the induced lightning types as follows:

K1. The overvoltage for tip of the blade and the tower top are not affected by the location at which the lightning hits. That is logical as these are the first points to receive the strike and network parameters have not been into action at this point.
K2. Negative strikes had shown to induce higher overvoltages than other types inside the grid.
K3. The injected overvoltage reaching the grid transformer are of highest magnitude when strike hits farthest tower from the grid for positive, MSC and MSS strikes as shown in cases R-2, R-6 and R-8.
K4. The overvoltage at the grid transformer are highest when strike hits closest tower to the grid for negative strikes as shown in cases R-3.

Previous features will be further discussed and analyzed in section 5. But within this section, the remaining topologies are tested to see if these features apply to them or not, and more importantly to compare which topology allows the least overvoltage to be injected to the grid.

### Single sided ring topology

**Fig 9:**
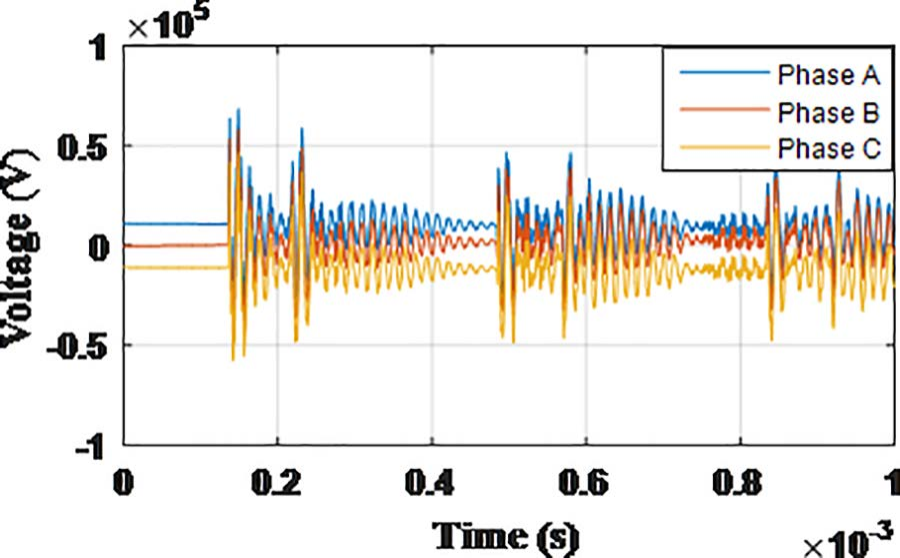
Overvoltages at low-voltage bus of the grid connected transformer in SSR topology for case SSR-1.

**Fig 10:**
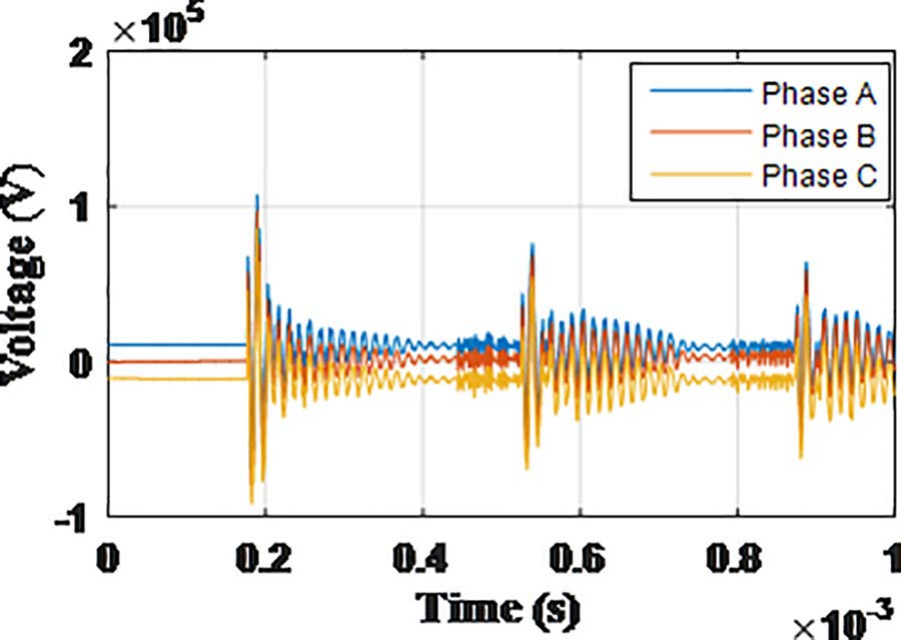
Overvoltages at low-voltage bus of the grid connected transformer in SSR topology for case SSR-2.

**Fig 11:**
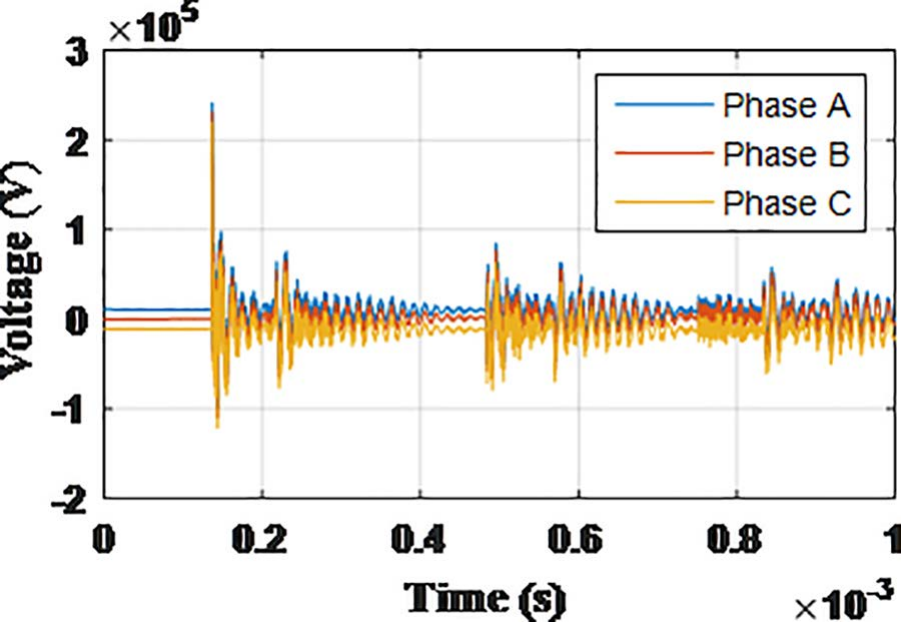
Overvoltages at low-voltage bus of the grid connected transformer in SSR topology for case SSR-3.

**Fig 12:**
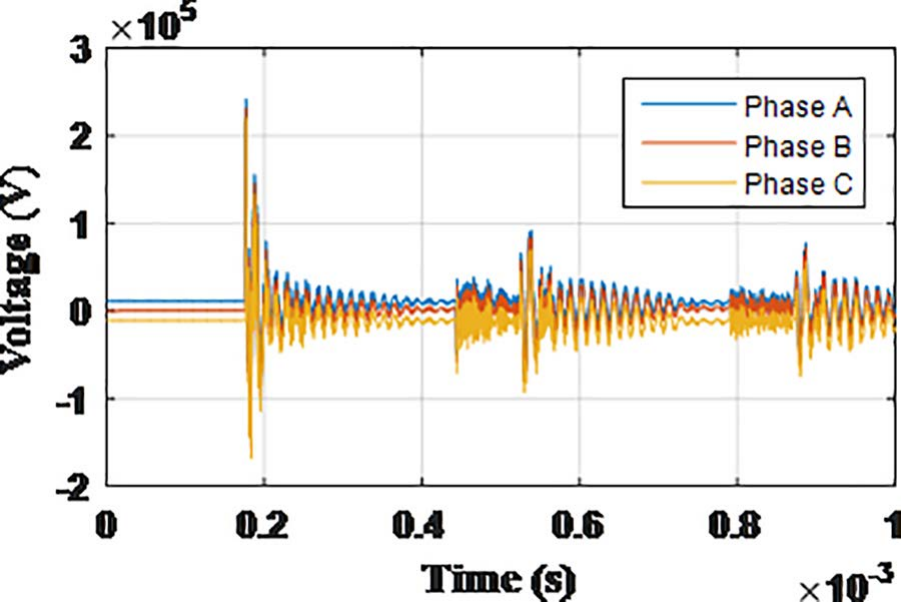
Overvoltages at low-voltage bus of the grid connected transformer in SSR topology for case SSR-4.

**Fig 13:**
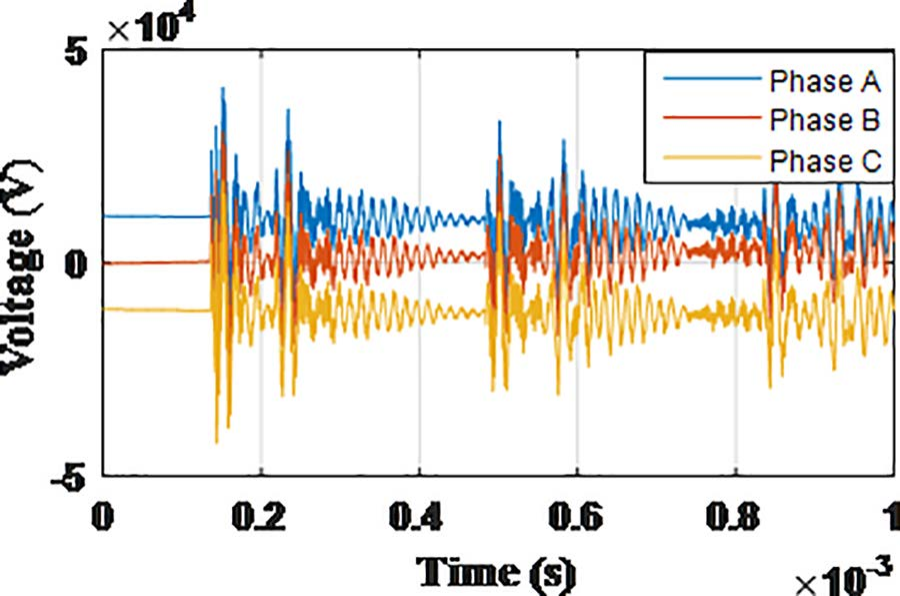
Overvoltages at low-voltage bus of the grid connected transformer in SSR topology for case SSR-5.

**Fig 14:**
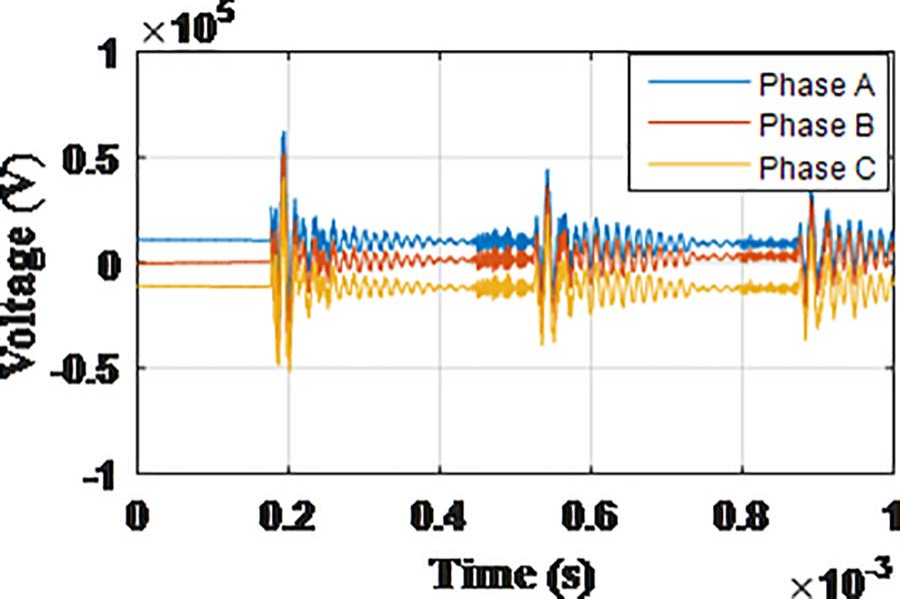
Overvoltages at low-voltage bus of the grid connected transformer in SSR topology for case SSR-6.

**Fig 15:**
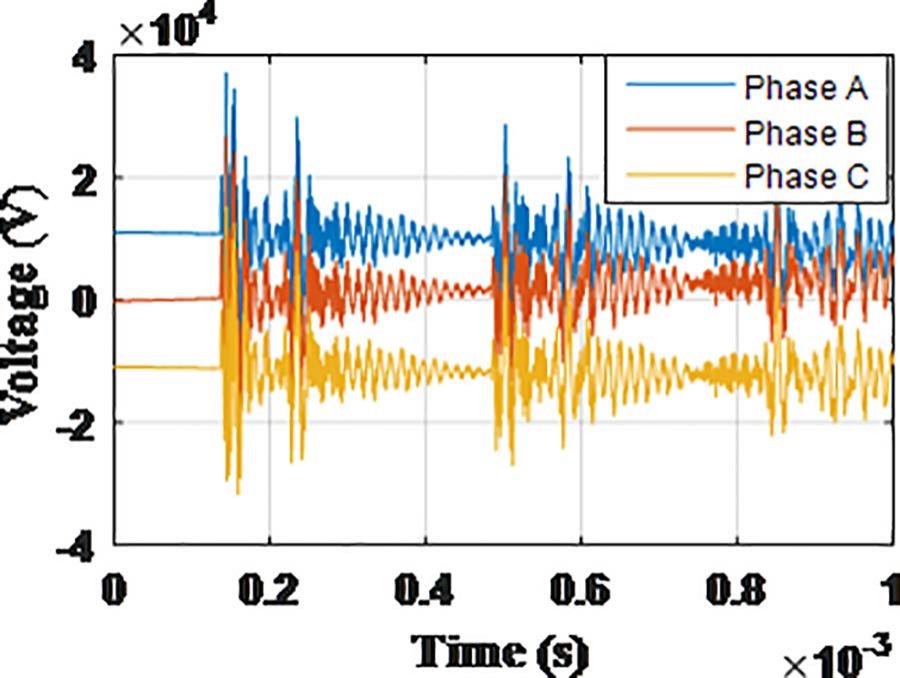
Overvoltages at low-voltage bus of the grid connected transformer in SSR topology for case SSR-7.

**Fig 16:**
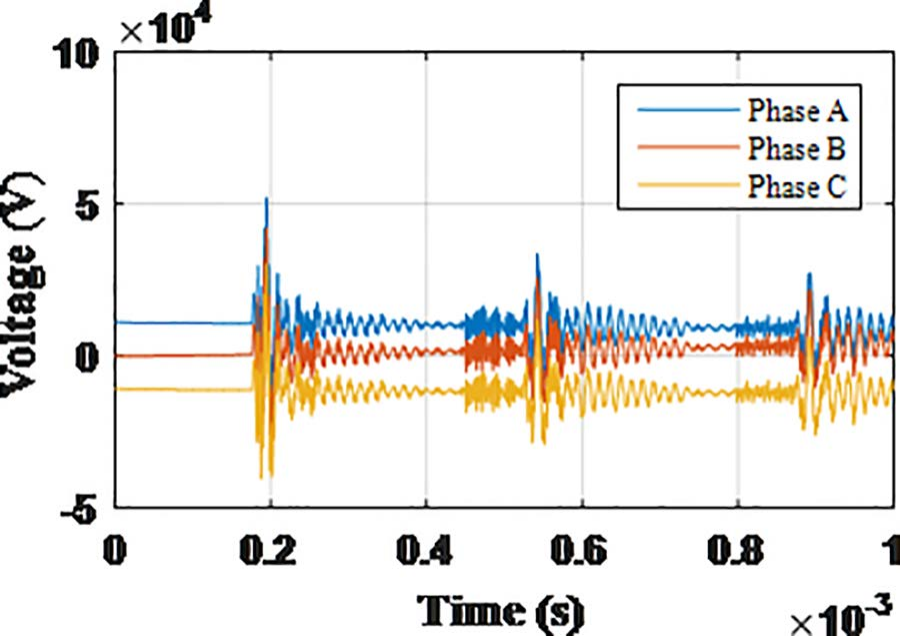
Overvoltages at low-voltage bus of the grid connected transformer in SSR topology for case SSR-8.

The same sequence of cases previously used, will be used in this topology. The notation for cases in this topology will be given initial SSR so case 1 where 50 kA, 10/350 µs positive lightning strike hits W1 will be named as SSR-1. For second case, where same lightning type as first case hits W13 will be named as SSR-2. The remaining cases are detailed in Table 4 which shows the maximum overvoltage recorded for each case. The maximum overvoltage at the low voltage bus of the grid transformer for case SSR-1 to SSR-8 are shown in Fig 9–16.

**Table 4:** Maximum overvoltages recorded for SSR topology.

Case name	Turbine hit	Type and parameters of lightning	Maximum Voltage at (MV)			Maximum Voltage at low voltage bus of grid-connected transformer (kV)		
			blade	Top of tower	Generator	Phase A	Phase B	Phase C
SSR- 1	W1	Positive 50 kA, 10/350 µs	6.11	3.54	1.12	68.51	58.12	57.52
SSR- 2	W13	Positive 50 kA, 10/350 µs	6.11	3.54	1.24	107.29	97.15	91.34
SSR- 3	W1	Negative 50 kA, 1/200 µs	6.69	4.41	1.39	241.54	231.08	219.58
SSR- 4	W13	Negative 50 kA, 1/200 µs	6.69	4.41	1.39	241.92	231.7	219.96
SSR- 5	W1	MCS, parameters from table 1	3.85	2.39	0.72	41.19	30.81	42.23
SSR- 6	W13	MCS, parameters from table 1	3.85	2.39	0.72	62.41	52.31	51.62
SSR- 7	W1	MSS, parameters from table 1	3.23	1.72	0.49	37.16	26.74	31.75
SSR- 8	W13	MSS, parameters from table 1	3.23	1.71	0.49	52.1	41.99	40.32

It is observable that the same key features describing the induced overvoltage that were earlier stated in section 4.1 for radial topology, also applies for SSR topology. Generally, when cases of SSR topology with their counterparts in radial topology, as SSR-1 compared to R-1, SSR-2 to R-2 …, it is obvious the SSR topology has a lower overvoltage compared to radial topology. The amount of reduction of the maximum overvoltage between SSR and radial topology is calculated by subtraction of the recorded values between Tables 3 and 4 and divided by that of Table 3 to get the percentage of reduction with respect to original radial topology. The reduction percentage ranged from 11.52% for phase A from SSR-4 when compared to R-4, to 51.03% for phase C of SSR-3 when compared to R-3. Hence, additional key feature is added to those mentioned in section in section 4.1 as follows:

K5. Induced lightning overvoltage reaching the transformer connecting to the grid are generally reduced in SSR topology than radial topology by range from 11.52% to 51.03%.

### Double sided ring topology

The sequence of testing is repeated for the double sided ring topology. The naming of testing cases will be in same manner as in previous topologies with initials DSR for each case. The maximum recorded overvoltage at each case is presented in Table 5. The results show the continuation of applicability of the extracted key characteristics for induced lightning overvoltages mentioned previously upon this topology. In addition, the results for maximum overvoltages in the DSR topology are much lower than in radial topology. The percentage of reduction of the maximum overvoltage recorded could be computed by subtracting results of Tables 3 and 5 and dividing by the values in Table 3 as used in previous section. The reduction percentage were from 39.5% for phase C in DSR-7 to 66.03% for phase B in DSR-8. Hence, key characteristic K5 also applies for DSR topology with even higher reduction percentage than SSR.

**Table 5:** Maximum overvoltages recorded for DSR topology.

Case name	Turbine hit	Type and parameters of lightning	Maximum Voltage at (MV)			Maximum Voltage at low voltage bus of grid-connected transformer (kV)		
			blade	Top of tower	Generator	Phase A	Phase B	Phase C
DSR- 1	W1	Positive 50 kA, 10/350 µs	6.11	3.54	1.24	68.28	57.76	57.21
DSR- 2	W13	Positive 50 kA, 10/350 µs	6.11	3.54	1.24	71.17	63.46	75.07
DSR- 3	W1	Negative 50 kA, 1/200 µs	6.67	4.41	1.39	240.62	230.02	218.78
DSR- 4	W13	Negative 50 kA, 1/200 µs	6.67	4.41	1.39	99.91	98.99	110.57
DSR- 5	W1	MCS, parameters from table 1	3.85	2.37	0.71	41.05	30.7	41.98
DSR- 6	W13	MCS, parameters from table 1	3.85	2.37	0.71	41.76	34.48	46.1
DSR- 7	W1	MSS, parameters from table 1	3.22	1.71	0.48	37.05	26.5	31.54
DSR- 8	W13	MSS, parameters from table 1	3.22	1.71	0.48	35.86	25.84	37.45

### Star topology

**Fig 17:**
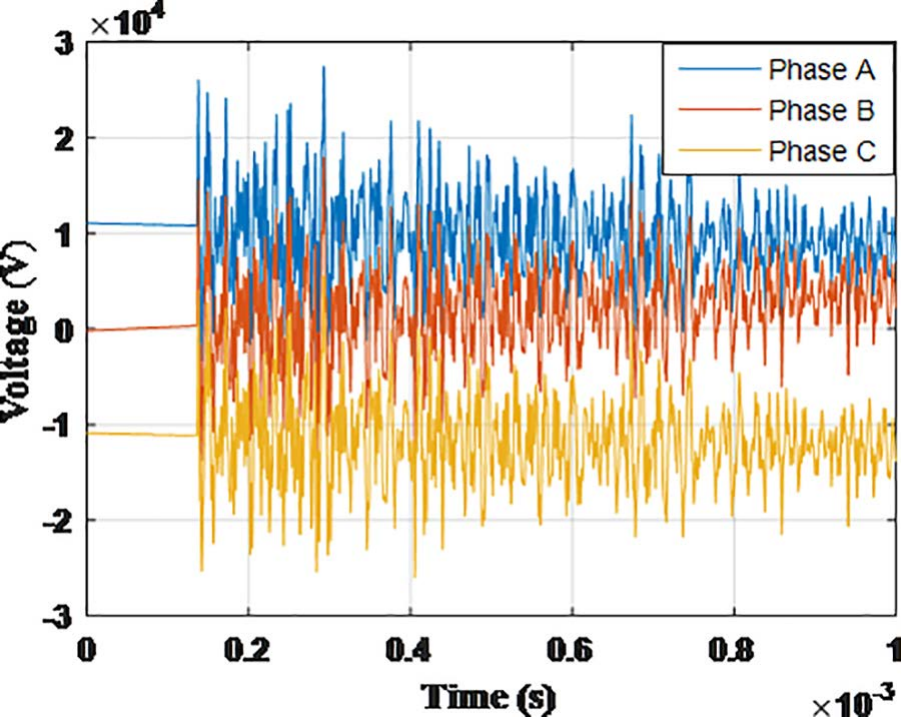
Overvoltages at low-voltage bus of the grid connected transformer in SSR topology for case S-1.

**Fig 18:**
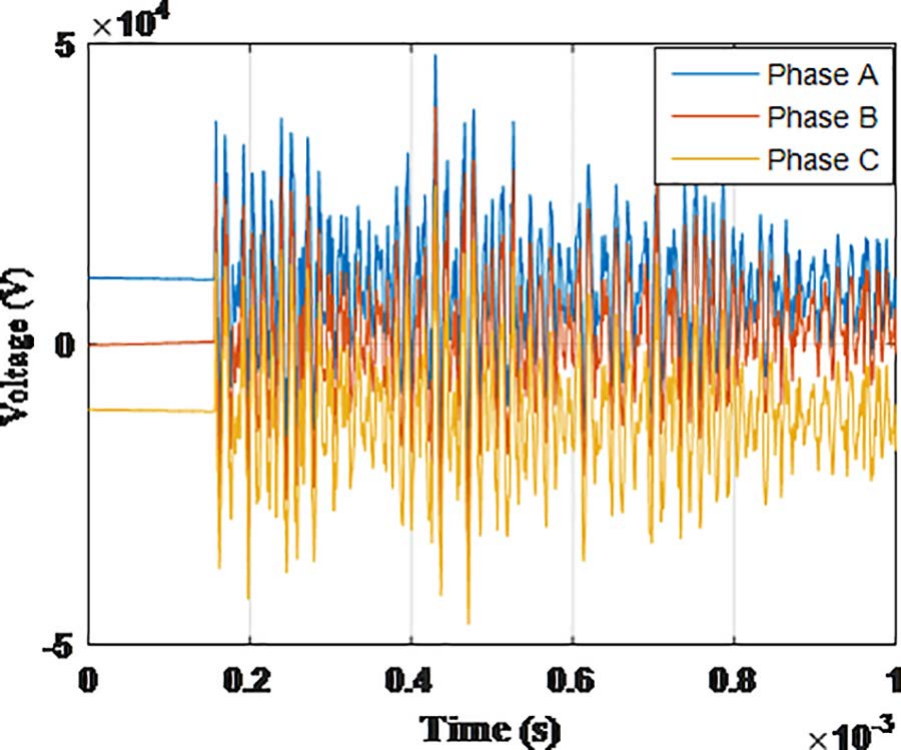
Overvoltages at low-voltage bus of the grid connected transformer in SSR topology for case S-2.

**Fig 19:**
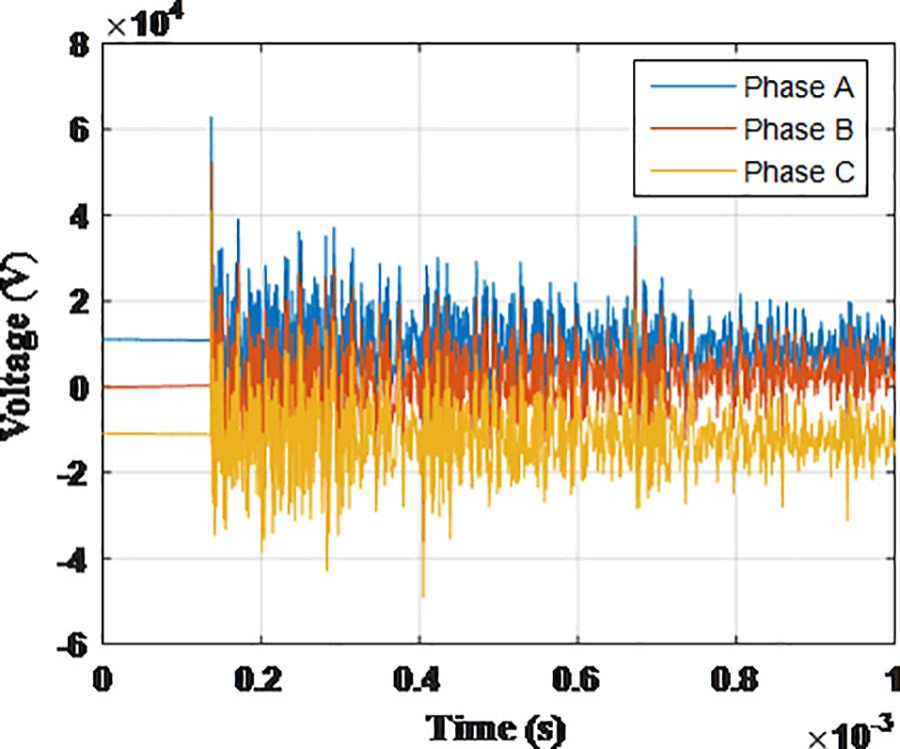
Overvoltages at low-voltage bus of the grid connected transformer in SSR topology for case S-3.

**Fig 20:**
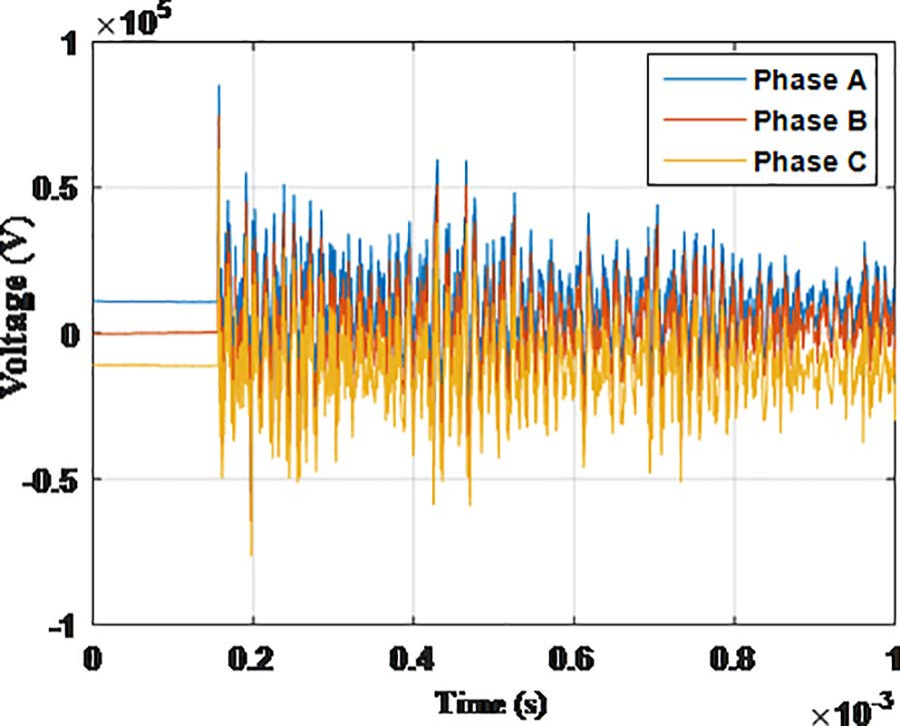
Overvoltages at low-voltage bus of the grid connected transformer in SSR topology for case S-4.

**Fig 21:**
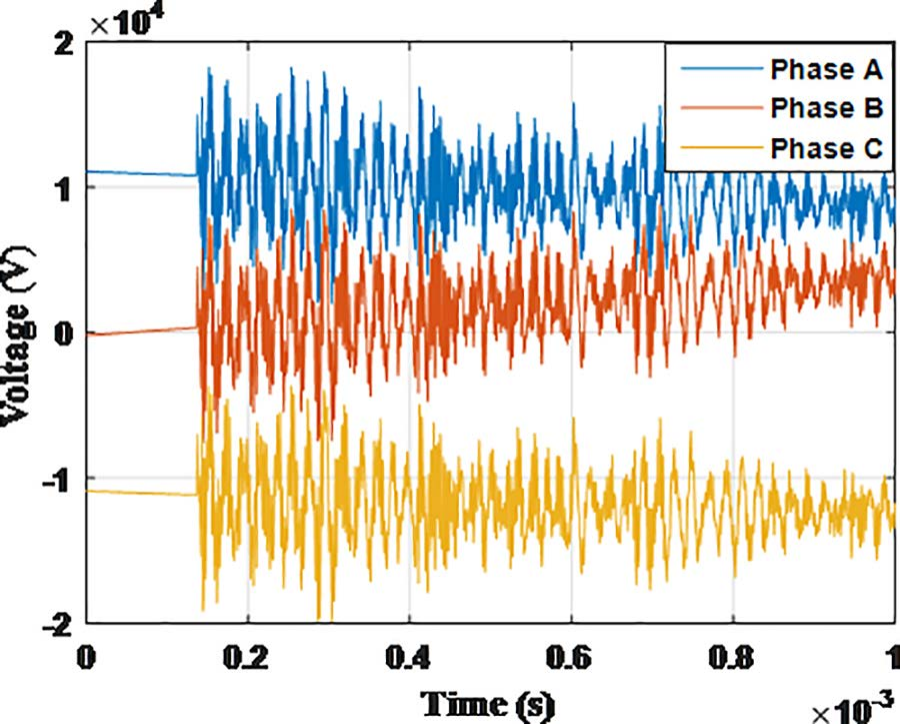
Overvoltages at low-voltage bus of the grid connected transformer in SSR topology for case S-5.

**Fig 22:**
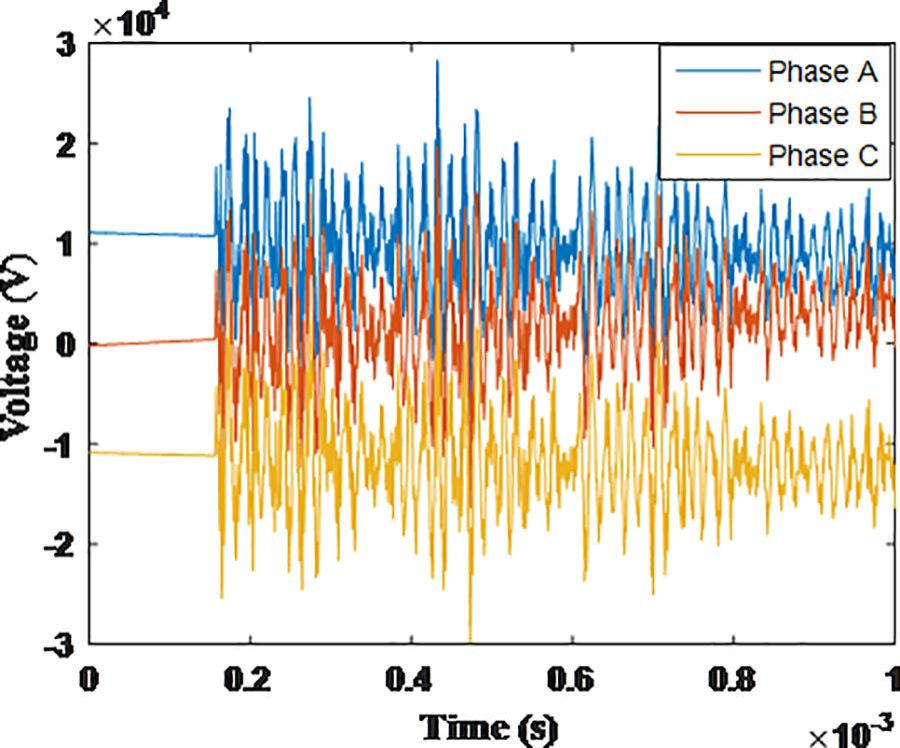
Overvoltages at low-voltage bus of the grid connected transformer in SSR topology for case S-6.

**Fig 23:**
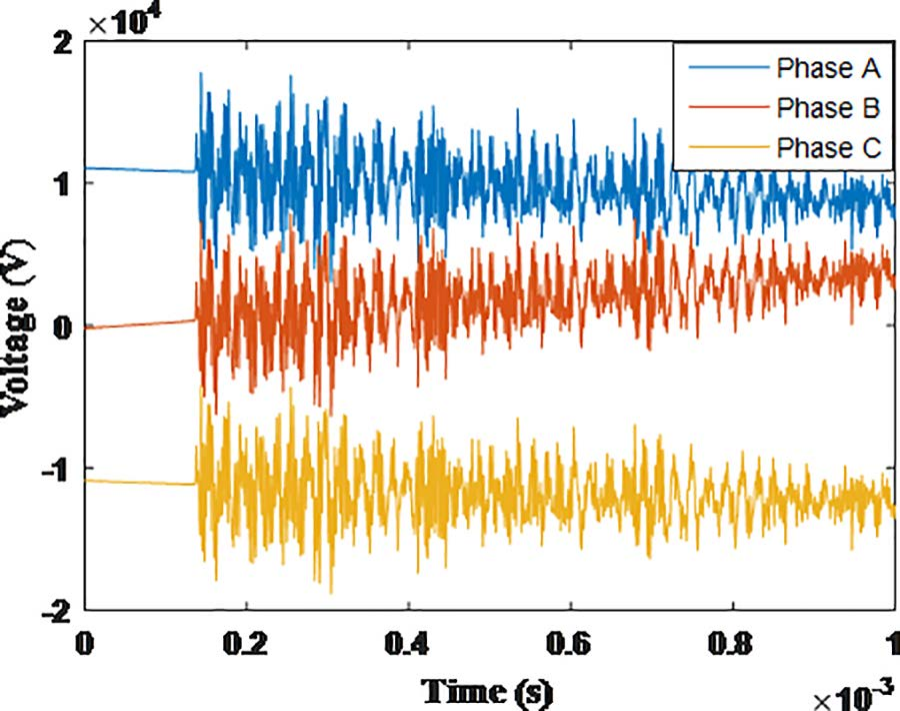
Overvoltages at low-voltage bus of the grid connected transformer in SSR topology for case S-7.

**Fig 24:**
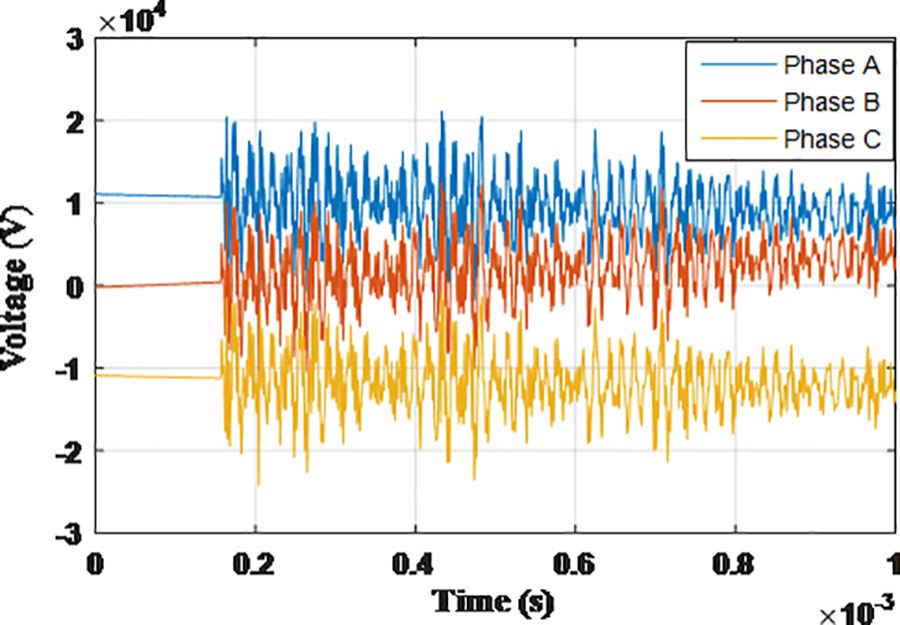
Overvoltages at low-voltage bus of the grid connected transformer in SSR topology for case S-8.

The sequence of testing is re-applied to the final topology. The notation guide will be in the same manner with initial letter S given to cases so they are numbered from S-1 to S-8. The maximum recorded overvoltage is presented in Table 6. The overvoltage recorded at the low voltage side of the transformer connected to the grid for cases S1 to S8 are shown in Figs 17–24. The results show the compliance of the start topology results with key features K1 to K4 previously mentioned. It is also observable that star topology injects the minimal overvoltage in comparison to SSR, DSR and radial topologies. The percentage of reduction in the maximum reordered overvoltage was also calculated in the same manner as in previous sections. It ranged from 62.25% for phase C in case S-8 to 89.04% for phase C in case S-3. It is evident that the star topology has the highest percentage of reduction of overvoltage with respect to radial, SSR and DSR topologies. Therefore, K5 key characteristic could be extended to include the star topology has highest percentage of reduction for grid-injected overvoltage. The key characteristic K1 to K5 will be analyzed and explained within the next section.

**Table 6:** Maximum overvoltages recorded for star topology.

Case name	Turbine hit	Type and parameters of lightning	Maximum Voltage at (MV)			Maximum Voltage at low voltage bus of grid-connected transformer (kV)		
			blade	Top of tower	Generator	Phase A	Phase B	Phase C
S- 1	W1	Positive 50 kA, 10/350 µs	6.11	3.56	1.44	27.43	17.93	25.98
S- 2	W13	Positive 50 kA, 10/350 µs	6.11	3.53	1.11	48.17	39.54	46.59
S- 3	W1	Negative 50 kA, 1/200 µs	6.69	4.49	1.51	62.84	52.48	49.13
S- 4	W13	Negative 50 kA, 1/200 µs	6.69	4.35	1.31	85.21	74.88	76.24
S- 5	W1	MCS, parameters from table 1	3.85	2.41	0.74	18.25	13.58	19.88
S- 6	W13	MCS, parameters from table 1	3.85	2.38	0.69	28.28	19.66	29.8
S- 7	W1	MSS, parameters from table 1	3.22	1.74	0.51	17.78	13.25	18.83
S- 8	W13	MSS, parameters from table 1	3.23	1.71	0.47	21.14	13.85	24.24

## Analysis and discussion of simulation results

**Fig 25:**
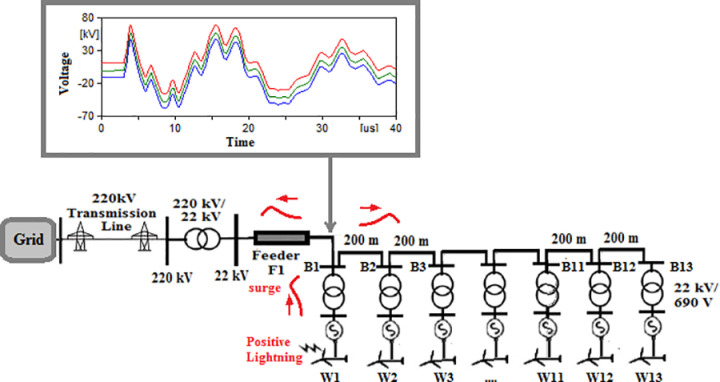
Propagation of overvoltage surges in radial topology when positive strikes hit W1.

**Fig 26:**
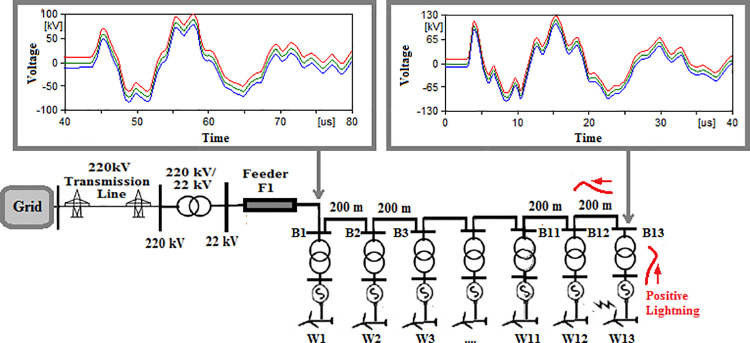
Propagation of overvoltage surges in radial topology when positive strikes hit W13.


In this section, key features extracted from results of previous section are discussed and explained based upon electrical engineering fundamentals. Each feature is explained in the following order;

**K1**: This characteristic is straightforward and requires no further explanation, as it indicates that the overvoltage formed at the tip of the blade and the top of the tower is unaffected by the wind farm topology. When lightning strikes the blade, the primary factors influencing the formation of the overvoltage are the type of lightning strike and the impedances of the blade and tower. Therefore, at this stage, the wind farm topology does not play a role in the overvoltage generated at the blade.
**K2:** The characteristics of lightning-induced overvoltages reveal that negative lightning strikes inject higher overvoltages into the grid compared to other types of lightning, even when positive lightning strikes have the same 50 kA peak as negative strikes. To better understand this, it’s important to consider the flow path of the lightning surge. When lightning strikes the blade, a surge propagates through the tower to the ground, inducing an overvoltage through capacitive coupling into the generator circuit within the turbine. The magnitude of the induced overvoltage is influenced by the rate of change of flux of the original lightning surge. Consequently, the injected overvoltage depends on the rise time and tail duration of the lightning strike. According to the IEC 61400-24 standard [39], negative strikes have a rise time of 1 µs, which is much faster than the 10 µs rise time of positive strikes [39], as well as the rise times of double-peaked strikes (Table 1). As a result, negative strikes have the highest rate of change of current and flux among the various lightning types, which leads them to induce the highest overvoltage into the grid.
**K3:** discusses that overvoltage injected the grid connected transformer due to positive, MCS and MSS strikes are highest when these strikes hit the farthest turbine rather than the closest. Even though this point might seem in contrast to the expected, but it could be analyzed as follows. Consider the radial topology of Fig 1 that is extended for explanation in this point in Figs 25 and 26. For a positive strike hitting blade of W1, the lightning induces overvoltage into the generator circuit which propagates as surge. At bus B1 shown in Fig 25, the surge has two flow paths a head, one downward to the remaining turbines and the second forward to the feeder F1 and the transformer. The surge acts now as a traveling wave which at each connection point part of it gets transmitted and part gets reflected. The transmission coefficient is given as [42]

$$T\;=\frac{2\;Z_{2}}{Z_{1}+\;Z_{2}}$$ (7)

Where $Z_{2}$ is the impedance of the line to which the surge is transmitted two and $Z_{1}$ is the impedance of the line from which the wave is incident. So the surge at bus B1, gets transmitted to both feeder F1 and cable connected to W2.

On the second situation, lightning hits W13 which induces overvoltage into the generator of that turbine. The injected overvoltage reaches bus B13 with no interconnection points as in previous case. Thus, avoiding the division of surge among different paths and leading to higher magnitude for the surge at bus B13 as shown in Fig 26. It is obvious the injected voltage at bus B13 in this case is higher than the injected overvoltage in previous case at bus B1 of Fig 25. The surge at bus B13 propagates upward through the farm till reaches bus B1 thus losing portion of its magnitude in the process. However, the surge reaching bus B1 in this case is still higher than that at B1 from Fig 25. This is explained for two reasons; first the cables are of equal length leading to maximum transfer of surge from one cable to another. Secondly, the transformer connected to turbines W12 to W1 have impedance dependent on their frequency, and since positive strikes have large rise time, their expected frequency for transformers will be low and so is their impedance. Hence, minimal parts of surge are going to deviated to the transformers with low impedances according to (7). Therefore, most of the original surge flowing from bus B13 will reach bus B1 as cables allowed maximum transfer of surge and transformer did not absorb any Signiant amount of the surge magnitude. Thus to summarize this part, lightning strikes hitting far turbines from the grid lead to higher injected overvoltage than close ones for two main reasons.

1. The surge hitting the far turbine had only one path to flow through at the hitting location avoiding any divisions in the surge.
2. The transformers connected to turbines W12 to W1 on the path of surge absorbed minimal amount of the surge due to their relative low impedance for positive strikes.

**Fig 27:**
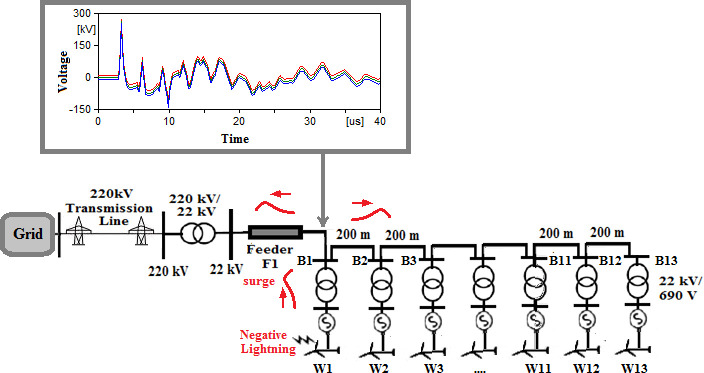
Propagation of overvoltage surges in radial topology when negative strikes hit W1.

**Fig 28:**
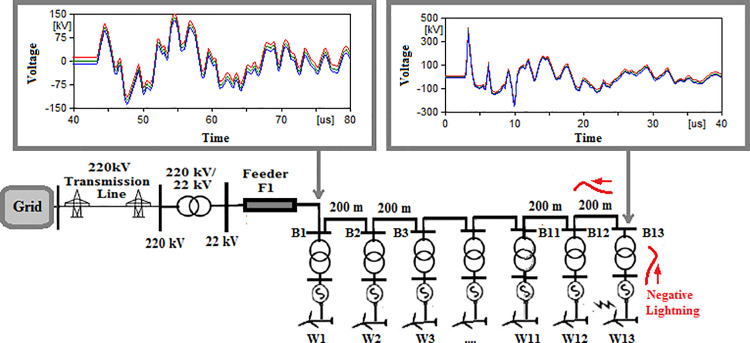
Propagation of overvoltage surges in radial topology when negative strikes hit W1.

**K4:** This characteristic is the reverse of the previous characteristic for negative strikes. The main reason for this change is that negative strikes have lower rise time than positive strikes as mention stated earlier. Thus, the frequency dependent impedances of transformers connected to the turbines will be relatively higher for negative strikes than other types. So consider the same cases where negative lightning strikes W1 in Fig 27 and second case where it strikes W13 in Fig 28. For case in Fig 27, the strike will propagate as explained in previous figure. For the second case, the negative lightning strikes W13 leading to higher overvoltage at bus B13 than that rising on bus B1 from previous case. This is because at bus B13 no interconnection is found, and most of the surge is obligated to flow to the path leading to bus B1 as explained in previous figure. However, as the surge propagates from bus B13 to bus B1, the transformers will have larger impedances for negative strikes allowing them to absorb larger parts of magnitude from the original surge according to (7). Thus the surge will be significantly reduced on its path from B13 to B1. It is observed that the overvoltage reaching bus B1 in Fig 28 is lower than that at 27. Thus, the main difference for negative strikes to differ in K4 from other strikes in K3, is the increase in the impedance of the transformer due to low rise time of negative strike.

**K5**: is the final and the most important characteristic within this study. It was observed that radial topology led to higher injected overvoltage into the grid with SSR and DSR having lower magnitudes in descending order respectively. The star topology had the least overvoltage injected to the grid. The percentage of reduction of overvoltage for SSR DSR and star topologies with respect radial topology were 11.52% to 51.03%, 39.5% to 66.03% and 62.25% to 89.04% respectively. It confirms that the star topology had the highest percentage of reduction in overvoltage compared to radial topology. This could be explained by the following reasons;

1. In star topology, the injected lightning surge is obligated to be divided among several branches regardless of the hitting location due to the star connection nature of the topology. This leads for the magnitude for injected surge to be divided among these paths.
2. As the amount of the connection branches gets lower in respective manner in DSR, SSR and radial topologies, the induced overvoltages becomes higher. This is due to the surges propagating through these topologies are no longer divided among as many branches as in star topology. That leads to larger surges to reach the grid.
3. The connecting cables in star topology are of different lengths. This creates an impedance mismatch, leading to reflection of parts of the lightning surge and reducing the amount of the transmitted surge to the grid. While for DSR, SSR and radial topologies, the cables used were of equal lengths that lead to higher transfer of lightning surges through them.

## Conclusion

This study investigated the risks posed by lightning strikes to wind farms, focusing on the impact of different wind farm topologies and lightning types. The primary goals were to extract the characteristic behavior of lightning-induced overvoltages in various wind farm configurations and assess how topology influenced the magnitude of these overvoltages. Using four wind farm topologies—radial, SSR, DSR, and star—based on data from a real system in Zaafrana, Egypt, and modeling the system with ATP/EMTP software, the study evaluated negative, positive, and double-peaked lightning strikes. The results revealed the following key characteristics:

**K1.** The lightning overvoltage at the tip of the blade at the top of the tower of the wind turbine was independent of the type of wind farm topology.
**K2.** The injected overvoltage due to negative strikes was higher than that of positive or double-peaked strikes, due to its steep rising front.
**K3.** The injected overvoltage to the grid when the lightning strike hit the farthest turbine from the grid was higher than when the strike hit the turbine closest to the grid for positive and double-peaked strikes.
**K4.** For negative strikes, the injected overvoltage to the grid when the lightning strike hit the closest turbine to the grid was higher than when the strike hit the turbine farthest from the grid.
**K5.** Radial topology exhibited the highest injected overvoltage. The use of SSR, DSR, and star topologies resulted in reductions in overvoltage, with a percentage reduction range of 11.52% to 51.03%, 39.5% to 66.03%, and 62.25% to 89.04% for each topology, respectively.

This study provided insights into lightning-induced overvoltages in wind farms and the role of topology in mitigating these risks. Based on the results, future recommendations for design include adopting the star topology to minimize overvoltage injection into the grid. Future research should focus on optimizing topological configurations with lightning protection strategies and investigating the long-term effects of overvoltages on grid stability and wind farm equipment.
